# Editorial: A focus on chaperone clients

**DOI:** 10.3389/fmolb.2023.1180739

**Published:** 2023-03-17

**Authors:** Amnon Horovitz, Abdussalam Azem

**Affiliations:** ^1^ Department of Chemical and Structural Biology, Weizmann Institute of Science, Rehovot, Israel; ^2^ School of Neurobiology, Biochemistry and Biophysics, The George S. Wise Faculty of Life Sciences, Tel Aviv University, Tel Aviv, Israel

**Keywords:** GroEL, chaperonin, hsp70, ClpP, chaperones, protein folding

Protein misfolding and aggregation are detrimental to cells owing to i) the absence (or decrease in number) of functional protein molecules that may have essential roles; ii) the energetic costs involved in the synthesis and degradation of non-functional proteins; and iii) the toxic effects of misfolded proteins or their aggregates (Stefani and Dobson, 2003). Consequently, the sequences of many proteins have evolved so that their energy landscapes are funneled in accordance with the principle of minimal frustration, thereby enabling them to fold rapidly to their native biologically functional conformations (Onuchic et al., 1997). Nevertheless, aggregation can still occur because of i) functional constraints on protein sequences and topologies that result in frustration and ii) conditions in the cell, such as crowding, which can favor aggregation. Various machineries for preventing or reversing protein aggregation and removal of aggregates have, therefore, evolved that include different types of molecular chaperones, disaggregases and proteases. The focus of this Research Topic is on the molecular and evolutionary mechanisms that govern the interactions of substrate (client) proteins with such machineries.

Many chaperone families have multiple clients but still display either broad or narrow specificity in their interactions with clients. A key question that arises, therefore, concerns the features that distinguish clients from non-clients. DapA and YagE, for example, are two *Escherichia coli* proteins with high sequence similarity and almost identical structures but the former is a GroE chaperonin client whereas the latter is not (Kerner et al., 2005). Likewise, protein homologs often differ in their interaction with GroE. For example, *E.coli* and mouse dihydrofolate reductases are weak and strong interactors with GroE, respectively (Clark and Frieden, 1997). The GroE system comprises GroEL and its co-factor GroES and assists protein folding in an ATP-dependent manner (Weiss et al., 2016; Balchin et al., 2020; Horovitz et al., 2022). The structural and biological basis of client specificity in this system are discussed in this Research Topic by Taguchi and Koike-Takeshita and Stan et al.

Molecular chaperones also impact client evolution in a variety of ways. It was suggested, for example, that chaperones such as hsp90 (Queitsch et al., 2002) and GroEL (Tokuriki and Tawfik, 2009) can buffer deleterious mutations, thereby promoting genetic variation and evolution. Molecular chaperones can also impact horizontal gene transfer and virus (or phage)-host interactions. Some phage proteins, for example, rely on host chaperonins for their folding (Hildenbrand and Bernal, 2012). Other infectious organisms, however, contain their own chaperonin systems. In this Research Topic, Wilkinson et al. describe the identification and analysis of the interactome of the eukaryotic chaperonin containing TCP-1 (CCT/TRiC) from the human malarial parasite, *P. falciparum*.

Another major and ubiquitous chaperone family is hsp70, members of which function in cooperation with co-chaperones of the J-domain protein (JDP) family (also referred to as DnaJ, hsp40) and nucleotide exchange factors (Mayer and Gierasch, 2019; Rosenzweig et al., 2019; Balchin et al., 2020). Both prokaryotic and eukaryotic genomes contain multiple variants of hsp70 and J-domain proteins. Humans, for example, contain 13 hsp70 homologues, which are expressed in distinct cellular compartments. One important question concerns identifying the different client specificities of the various hsp70 and J-domain proteins. An example for a very specific J-domain protein is hsc20, which is involved in FeS cluster biogenesis in both prokaryotes and eukaryotes. Its only known client is the Isu/IscU scaffold on which the FeS clusters are built before being transferred to other proteins. Hsc20 can function with either a specialized or multi-functional hsp70. This Research Topic contains a review by Marszalek and Craig of this specialized hsp70 system.

Proteolytic machineries provide a back-up solution for eliminating protein aggregates when their formation was not prevented or reversed with the aid of chaperones (Sauer and Baker, 2011). One such machinery found in prokaryotes is ClpXP, which consists of an ATP-dependent protein unfoldase and translocase (ClpX) and a protease (ClpP). ClpP consists of two heptameric rings that form a proteolytic chamber. Given that aggregates impair fitness, inhibiting ClpXP provides a potential strategy for fighting bacterial infections as discussed in this Research Topic by Aljghami et al.

In summary, this Research Topic highlights that to understand the functions of molecular chaperones it is not enough to determine their structures and reaction cycles. It is also essential to know how clients are selected and recognized and establish the mechanisms by which clients and chaperones co-evolve.
